# Anemia Profiles in Cancer Patients: Prevalence, Contributing Factors, and Insights From a Retrospective Study at a Single Cancer Center in Saudi Arabia

**DOI:** 10.7759/cureus.42400

**Published:** 2023-07-24

**Authors:** Ahmed M Badheeb, Faisal Ahmed, Mohamed A Badheeb, Hamoud Y Obied, Islam A Seada, Abdulaziz Al Jumman, Nasher H Alyami, Musadag Elhadi, Abbas H Almakrami, Ibrahim Mokhtar

**Affiliations:** 1 Oncology, King Khalid Hospital, Najran, SAU; 2 Urology, Ibb University, Ibb, YEM; 3 Internal Medicine, Yale New Haven Health, Bridgeport Hospital, Bridgeport, USA; 4 Cardiac Surgery, King Khalid Hospital, Najran, SAU; 5 Cardiothoracic Surgery, King Khalid Hospital, Najran, SAU; 6 Internal Medicine, King Khalid Hospital, Najran, SAU; 7 Hematology, Ministry of Health Holdings, Najran, SAU; 8 Critical Care, King Khalid Hospital, Najran, SAU; 9 Endocrinology, King Khalid Hospital, Najran, SAU; 10 Diabetes and Endocrinology, King Khalid Hospital, Najran, SAU

**Keywords:** saudi arabia, associated factors, prevalence, cancers, chemotherapy, anemia

## Abstract

Background: Anemia, a common complication of cancer and its treatments, significantly affects cancer patients’ survival and quality of life. Nevertheless, there is limited research conducted in the southern region of Saudi Arabia regarding its effects. This study aims to assess the prevalence of anemia, as well as its associated factors, among cancer patients undergoing active chemotherapy treatment.

Method: This retrospective study analyzed adult cancer patients who underwent chemotherapy at King Khaled Hospital's oncology department in Najran, Saudi Arabia, between 2017 and 2022. We aimed to determine the prevalence and contributing factors of anemia through comprehensive demographic and clinical assessment. Univariate analysis was performed to assess factors necessitating blood transfusion.

Result: A total of 95 cancer patients received chemotherapy, with a mean age of 52.2 ± 16.5 years. The majority were females (65.3%) aged between 18 and 64 years (74.7%). Gastrointestinal (42.1%) and breast (17.9%) cancers were the most prevalent malignancies. Most patients (56.8%) were in locally advanced stages. Anemia was present at admission in 48 (50.5%) patients with a higher prevalence among colorectal and genitourinary tract cancer patients. The mean hemoglobin (Hb) drop during treatment was 9.1 ± 2.1 g/dL. Anemia severity was stratified as follows: life-threatening (7.4%), severe (33%), moderate (31%), and lower limited (29%). Blood transfusions were required in 79% of cases. Advanced age, increased chemotherapy cycles, and anemia of chronic disease (ACD) were significantly associated with increased anemia severity (p<0.05). Increasing chemotherapy cycles also correlated with an increased need for blood transfusion (p<0.001). Older patients (≥65 years) had higher anemia at admission, poor Eastern Cooperative Oncology Group (ECOG) performance status, more Hb decrease during treatment, and increased need for blood transfusions (p<0.05) compared to younger patients (<65 years).

Conclusion: The study noted a high prevalence of anemia (50.5%) in patients receiving active cancer treatment, specifically in the context of genitourinary and gastrointestinal tract cancers. Advanced age, frequent chemotherapy cycles, and ACD were associated with increased severity of anemia. Furthermore, older patients displayed a higher frequency of anemia, poorer performance status, and an increased requirement for transfusions with an escalating number of chemotherapy cycles.

## Introduction

Cancer continues to emerge as a prominent global cause of mortality [1]. The incidence and death rates have witnessed a worrisome upward trajectory, with more than 19 million new cases and 609,820 deaths reported in 2023. This reflects a significant increase of more than 5 million cases and 2 million deaths compared to the statistics reported in 2012 [2,3]. Unfortunately, these figures are projected to escalate further to an estimated 23.6 million new cases by 2030 [3]. Despite continuous research endeavors, improved healthcare accessibility, and the advent of innovative treatment modalities, the incidence of cancer persistently rises [4]. This can be partly attributed to aging populations, prolonged exposure to carcinogenic substances, and various socioeconomic factors [1,3]. While chemotherapy has indisputably demonstrated clinical benefits in limiting cancer progression and inducing its remission, it is not devoid of risks and adverse events [5]. These adverse effects encompass various toxicities that can manifest acutely or chronically, affecting systems such as the gastrointestinal, cardiac, and neurological systems. Additionally, chemotherapy-induced myelosuppression presents deleterious effects on the bone marrow, impacting all cellular lineages [6].

Notably, chemotherapy-induced anemia (CIA) has recently received heightened attention. Pooled evidence indicates that anemic cancer patients experience worse outcomes and poorer survival rates [7]. Furthermore, optimizing the hematological profile of cancer patients has been proposed as a means to enhance their quality of life while simultaneously reducing healthcare expenses [8]. Previous research endeavors have aimed to identify significant determinants of CIA, including initial hemoglobin (Hb) levels, BMI, body surface area, age, glomerular filtration rate, the utilization of specific myelotoxic drugs (such as taxanes, high-dose anthracyclines, platinum compounds, or gemcitabine-based chemotherapy), and gender disparities [9-11]. Despite the critical role of CIA in cancer outcomes, it has not received adequate consideration as a prevalent and detrimental issue among cancer patients. To date, comprehensive studies assessing the optimal approach, risk classification and stratification, and therapeutic targets for CIA remain lacking [9,12].

In light of the previously reported elevated incidence rates and the limited research conducted in the specific region, our study aimed to bridge this knowledge gap and make a significant contribution to healthcare advancements at King Khaled Hospital, Najran City, Saudi Arabia (KKHN). Our primary focus was to examine and evaluate the prevalence of anemia among cancer patients undergoing active chemotherapy treatment. Through this investigation, we sought to bring attention to this urgent matter and provide valuable insights that can improve the monitoring and management of patients.

## Materials and methods

Study design

This study was conducted within the oncology department of KKHN and involved a retrospective chart review of chemotherapy recipients for a period of five years from Jun 2017 to October 2022. The ethical approval for the study was obtained through the Institutional Review Board of KKHN (H-l1-N-081), adhering to the ethical principles of the Helsinki Declaration.

Inclusion criteria

All adult cancer patients who were pathologically confirmed and treated with chemotherapy in our center and complete clinical data and follow-up information were included.

Exclusion criteria

This study excluded cancer patients who were not recipients of chemotherapy, individuals who missed their follow-up appointments, or those who underwent surgical procedures.

Data collection

Data regarding patient demographics such as age and gender, tumor pathological type, site of cancer, stage, history of cardiac illness, history of iron supplement consumption, ECOG performance status, history of previous bleeding, endoscopy and colonoscopy records, palliative care received, treatment modalities, chemotherapy cycles number, Hb drop during treatment, need for blood transfusion, anemia treatment, and laboratory data including complete blood cell count, mean corpuscular volume (MVC), mean corpuscular Hb (MCH), total iron binding capacity (TIBC), Hb (g/dL), ferritin level, serum iron (mcg/dL), cobalamin (B12) (pg/mL), type of anemia, the trigger Hb level for initiating treatment were collected from the electronic medical records. A standardized data collection sheet was utilized for this purpose.

Outcome

The main outcomes of interest were the prevalence and severity of anemia and the identification of associated factors. Additionally, the correlation between the need for blood transfusion and the number of chemotherapy cycles was examined. A comparison between older (≥65 years) and middle-aged cancer patients (<65 years) was also conducted.

Definition

Anemia in this study was classified according to the National Cancer Institute Anemia Scale, which categorizes anemia into different grades based on Hb levels. Grade 0 represents normal ranges (12-16 g/dL for females, 14-18 g/dL for males). Anemia was graded as follows: mild "Grade 1" (10-12 g/dL for women, 10-14 g/dL for men), moderate "Grade 2" (8-10 g/dL), severe "Grade 3" (6.5-8 g/dL), life-threatening "Grade 4" (<6.5 g/dL), and "Grade 5" corresponds to death [13]. The drop in Hb was defined as the baseline Hb level minus the Hb level at first transfusion was collected from the electronic medical records.

Statistical analysis

The data analysis was conducted using SPSS Statistics version 22 (IBM Corp. Released 2013. IBM SPSS Statistics for Windows, Version 22.0. Armonk, NY: IBM Corp.). The study utilized both quantitative and qualitative data, for which means and standard deviations were used to present quantitative data, while frequencies and percentages were reported for qualitative variables. To confirm normality, the Smirnov-Kolmogorov test was employed. For comparing continuous data, independent samples t-test or Mann-Whitney test was utilized, while categorical variables were compared using chi-square or Fisher's exact test. In order to identify independent risk factors associated with mortality, univariate analysis was performed, and OR with 95% CI was reported to determine the effect size. A significance level of p <0.05 was chosen for statistical analysis.

## Results

Demographic characteristics

We included a total of 95 chemotherapy-recipient cancer patients, with a mean age of 52.2 ± 16.5 years (minimum: 18 years - maximum: 86 years). The majority of patients (74.7%) fell within the age range of 18-64 years, and most of them were female (65.3%). A history of anemia at admission was observed in 48 (50.5%) patients. Gastrointestinal (42.1%) and breast (17.9%) cancers were the most prevalent malignancies. A significant proportion of patients (56.8%) were in locally advanced stages. History of heart disease, bleeding, and previous endoscopy or colonoscopy were reported in 24.4%, 29.5%, and 37% of patients, respectively. Table 1 provides detailed demographic characteristics of the patients.

**Table 1:** Prevalence of anemia and its associated factors among cancer patients treated at KKHN from January 2017 to February 2022 (n = 95) ECOG: Eastern Cooperative Oncology Group

Characteristic	Subgroups	N (%)
Age (year)	Mean± SD	52.2 ± 16.5 (min:18 - max:86)
	Between 18-64 years	71 (74.7)
	65 years or more	24 (25.3)
Gender	Male	33 (34.7)
	Female	62 (65.3)
History of anemia at admission	Yes	48 (50.5)
	No	47 (49.5)
Main symptoms	Fatigue	38 (40)
	Palpitations	24 (25.3)
	None	17 (17.9)
	Dizziness	16 (16.8)
Site of cancer	Gastrointestinal cancer	40 (42.1)
	Breast cancer	17 (17.9)
	Urologic cancer	14 (14.7)
	Hematologic cancer	6 (6.3)
	Head and neck cancer	5 (5.3)
	Sarcoma cancer	5 (5.3)
	Thyroid cancer	4 (4.2)
	Gynecological cancer	3 (3.2)
	Lymphoma	1 (1.1)
Cancer stage	Locally advanced	54 (56.8)
	Early stage	41 (43.2)
History of cardiac illness	-	23 (24.4)
Bleeding history	-	28 (29.5)
History of Iron supplement intake	-	16 (17)
History of darbepoetin alpha intake	-	54 (56.8)
ECOG performance status	Low Grades (0, 1, and 2)	51 (53.7)
	High Grades (3, 4, and 3)	44 (46.3)
History of endoscopy or colonoscopy	-	35 (37)

Laboratory data and needs for blood transfusion during chemotherapy treatment

The mean decrease in Hb (g/dL) during treatment was 9.1 ± 2.1 (minimum: 4.1, maximum: 15). Anemia was categorized as life-threatening (<6.5 g/dL) in seven (7.4%) cases, severe (6.5-8 g/dL) in 31 (33%) cases, moderate (8-10 g/dL) in 29 (31%) cases, and mild (≥10 g/dL) in 28 (29%) cases. MVC was low in 49 (52%) cases, while total serum iron binding capacity was high in 73% of cases. Serum iron (mcg/dL) was low in 46 (48%) cases, and serum ferritin (ng/mL) levels were high in 73 (77%) cases. ACD accounted for the majority of cases (74%). In Table 2, we summarized the laboratory findings of our patients.

**Table 2:** Laboratory data and blood transfusion need during cancer patients' treatments MCV: mean corpuscular volume, MCH: mean corpuscular hemoglobin, RDW: red cell distribution width, TIBC: total iron binding capacity, Hb: hemoglobin, ACD: anemia of chronic disease *Multimodalities include chemotherapy, radiotherapy, surgery, and/or hormonal therapies

Variables	Subgroups	N (%)
MCH	Normal	41 (43%)
	Low	47 (49%)
	High	7 (7.4%)
MCV	Normal	42 (44%)
	Low	49 (52%)
	High	4 (4.2%)
RDW%	Normal	20 (21%)
	High	75 (79%)
Hb (g/dL)	Mean± SD	9.1 ± 2.1 (in:4.1, max:15)
	Life-threatening (< 6.5)	7 (7.4%)
	6.5-8	31 (33%)
	8-10	29 (31%)
	≥10	28 (29%)
Serum Iron (mcg/dL)	low	46 (48%)
	High or NL	49 (52%)
TIBC (mcg/dL)	Low	25 (26%)
	Not done	1 (1.1%)
	High or normal	69 (73%)
Ferritin (ng/mL)	Low or normal	21 (22%)
	Not done	1 (1.1%)
	High	73 (77%)
Cobalamin (B12) (pg/mL)	Not done	1 (1.1%)
	High or NL	94 (99%)
Anemia type	ACD	70 (74%)
	Multi-factorial anemia	16 (17%)
	Iron def anemia	7 (7.4%)
	Megaloblastic anemia	2 (2.1%)
Blood transfusion during treatment	Yes	75 (79%)
	No	20 (21%)
Treatment modality	Chemotherapy	42 (44%)
	Immunotherapy and chemotherapy	6 (6.3%)
	Multimodalities*	47 (49%)
Number of chemotherapy cycle	<7	57 (60%)
	≥7	38 (40%)

Blood transfusions were required in 75 (79%) cases, with the majority of patients (60%) needing fewer than seven blood transfusions during the treatment period. The indication for blood transfusion, according to KKHN policy, was a Hb level lower than 7 g/dL to maintain Hb within (7-9 g/dL) range in 55.9% of cases. The interval between transfusions was less than one week in 37.5% of cases, between one and four weeks in 12.5% of cases, and more than one month in 25.0% of cases.

Comparison between older (≥65 years) and younger age (<65 years) cancer patients

We noticed that older cancer patients had a higher prevalence of a history of anemia at admission (p=0.039), were more likely to be in palliative care (p=0.009), had a poorer ECOG performance status (p=0.029), required more blood transfusions (p=0.011), and had more Hb decrease levels (g/dL) during treatment (p=0.020) compared to younger patients (<65 years). These differences were found to be statistically significant, as presented in Table 3.

**Table 3:** Comparison between old and middle age cancer patients ECOG: Eastern Cooperative Oncology Group, Hb: hemoglobin *p-values of < .05 were considered significant

Characteristic	Subgroups	Total	<65 years	≥65 years	p-value^*^
Gender	Male	33 (34.7)	22 (31.0)	11 (45.8)	0.283
	Female	62 (65.3)	49 (69.0)	13 (54.2)	
History of anemia at admission	No	47 (49.5)	40 (56.3)	7 (29.2)	0.039
	Yes	48 (50.5)	31 (43.7)	17 (70.8)	
Main symptom	None	17 (17.9)	12 (16.9)	5 (20.8)	0.339
	Palpitations	24 (25.3)	15 (21.1)	9 (37.5)	
	Dizziness	16 (16.8)	13 (18.3)	3 (12.5)	
	Fatigue	38 (40.0)	31 (43.7)	7 (29.2)	
Cancer site	Gynecological cancer	3 (3.2)	2 (2.8)	1 (4.2)	0.443
	Gastrointestinal cancer	40 (42.1)	30 (42.3)	10 (41.7)	
	Urologic cancer	14 (14.7)	11 (15.5)	3 (12.5)	
	Breast cancer	17 (17.9)	14 (19.7)	3 (12.5)	
	Lymphoma	1 (1.1)	0 (0.0)	1 (4.2)	
	Head and neck cancer	5 (5.3)	5 (7.0)	0 (0.0)	
	Hematologic cancer	6 (6.3)	3 (4.2)	3 (12.5)	
	Sarcoma	5 (5.3)	3 (4.2)	2 (8.3)	
	Thyroid cancer	4 (4.2)	3 (4.2)	1 (4.2)	
Cancer stage	Early stage	41 (43.2)	29 (40.8)	12 (50.0)	0.586
	Locally advanced	54 (56.8)	42 (59.2)	12 (50.0)	
Cardiac illness	No	72 (75.8)	53 (74.6)	19 (79.2)	0.864
	Yes	23 (24.2)	18 (25.4)	5 (20.8)	
Bleeding history	No	67 (70.5)	54 (76.1)	13 (54.2)	0.076
	Yes	28 (29.5)	17 (23.9)	11 (45.8)	
Palliative care	No	60 (63.2)	39 (54.9)	21 (87.5)	0.009
	Yes	35 (36.8)	32 (45.1)	3 (12.5)	
Iron intake	No	79 (83.2)	60 (84.5)	19 (79.2)	0.773
	Yes	16 (16.8)	11 (15.5)	5 (20.8)	
Darbepoetin alpha intake	Yes	54 (56.8)	43 (60.6)	11 (45.8)	0.307
	No	41 (43.2)	28 (39.4)	13 (54.2)	
ECOG performance status	Low grades	51 (53.7)	33 (46.5)	18 (75.0)	0.029
	High grades	44 (46.3)	38 (53.5)	6 (25.0)	
Number of chemotherapy cycle	Less than 7	57 (60.0)	46 (64.8)	11 (45.8)	0.162
	More than 7	38 (40.0)	25 (35.2)	13 (54.2)	
Number of transfusions	Mean (SD)	1.2 (2.0)	0.9 (1.4)	2.1 (3.0)	0.011
Lower Hb (g/dL) during treatment	Life-threatening (<6.5)	7 (7.4)	3 (4.2)	4 (16.7)	0.02
	6.5-8	31 (32.6)	22 (31.0)	9 (37.5)	
	08-Oct	29 (30.5)	27 (38.0)	2 (8.3)	
	More than 10	28 (29.5)	19 (26.8)	9 (37.5)	

Factors associated with anemia severity in chemotherapy recipients

The analysis demonstrated that older age (p=0.011), an increased number of chemotherapy cycles (p<0.001), and the presence of ACD (p=0.001) were significantly associated with greater severity of anemia during treatment. Detailed results are shown in Table 4.

**Table 4:** Factors associated with anemia severity in chemotherapy cancer patients ECOG: Eastern Cooperative Oncology Group, ACD: anemia of chronic disease *p-values of <0.05 were considered significant

Characteristic	<6 g/dL, 7 (7.4%)	6.5-8 g/dL, 31 (33%)	8-10 g/dL, 29 (31%)	≥ 10 g/dL, 28 (29%)	p-value*
Age group					0.011
18-64	3 (43%)	22 (71%)	27 (93%)	19 (68%)	
65 or more	4 (57%)	9 (29%)	2 (6.9%)	9 (32%)	
Gender					0.98
Male	2 (29%)	10 (32%)	11 (38%)	10 (36%)	
Female	5 (71%)	21 (68%)	18 (62%)	18 (64%)	
Anemia at admission	3 (43%)	13 (42%)	18 (62%)	14 (50%)	0.45
Cancer type					0.167
Gynecological cancer	1 (14%)	0 (0%)	1 (3.4%)	1 (3.6%)	
Gastrointestinal cancer	0 (0%)	14 (45%)	14 (48%)	12 (43%)	
Urologic cancer	1 (14%)	7 (23%)	2 (6.9%)	4 (14%)	
Breast cancer	2 (29%)	5 (16%)	7 (24%)	3 (11%)	
Lymphoma	0 (0%)	0 (0%)	0 (0%)	1 (3.6%)	
Head and neck cancer	1 (14%)	3 (9.7%)	0 (0%)	1 (3.6%)	
Hematologic cancer	0 (0%)	0 (0%)	3 (10%)	3 (11%)	
Sarcoma	2 (29%)	1 (3.2%)	1 (3.4%)	1 (3.6%)	
Thyroid cancer	0 (0%)	1 (3.2%)	1 (3.4%)	2 (7.1%)	
Cancer stage					0.268
Early stage	2 (29%)	10 (32%)	16 (55%)	13 (46%)	
Locally advanced	5 (71%)	21 (68%)	13 (45%)	15 (54%)	
Cardiac illness	2 (29%)	4 (13%)	9 (31%)	8 (29%)	0.31
Bleeding history	1 (14%)	13 (42%)	4 (14%)	10 (36%)	0.066
Palliative care	2 (29%)	13 (42%)	11 (38%)	9 (32%)	0.84
Iron intake	2 (29%)	4 (13%)	3 (10%)	7 (25%)	0.34
Darbepoetin alpha intake	5 (71%)	17 (55%)	16 (55%)	16 (57%)	0.91
ECOG performance status					0.56
Low (grades 0, 1, and 2)	3 (43%)	14 (45%)	17 (59%)	17 (61%)	
High (grades 3, 4, and 5)	4 (57%)	17 (55%)	12 (41%)	11 (39%)	
Number of chemotherapy cycle					<0.001
Less than 7	0 (0%)	0 (0%)	29 (100%)	28 (100%)	
More than 7	7 (100%)	31 (100%)	0 (0%)	0 (0%)	
Anemia type					0.001
ACD	2 (28.6)	17 (54.8)	27 (93.1)	24 (85.7)	
Multi-factorial anemia	3 (42.9)	11 (35.5)	1 (3.4)	1 (3.6)	
Iron def anemia	2 (28.6)	2 (6.5)	1 (3.4)	2 (7.1)	
Megaloblastic anemia	0 (0.0)	1 (3.2)	0 (0.0)	1 (3.6)	

Correlation between the need for blood transfusion and chemotherapy cycle numbers

Our analysis showed that with the increased number of chemotherapy cycles, the need for blood transfusion was increased (p <0.001), as shown in Table 5.

**Table 5:** Correlation between the need for blood transfusion and chemotherapy cycle numbers *p-values of <0.05 were considered significant

Number cycle	Blood transfusion (N=75)	No blood transfusion (N=20)	p-value^*^
Less than seven cycles	38.0 (50.7%)	19.0 (95.0%)	<0.001
More than seven cycles	37.0 (49.3%)	1.0 (5.0%)	

## Discussion

Anemia remains a significant and under-recognized issue among cancer patients, warranting attention and further investigation. The management of anemia in this specific population presents challenges due to the multifactorial nature of hematological abnormalities, which can arise from various factors including cancer progression, associated inflammatory processes, chemotherapy regimens, coagulopathies, bleeding disorders, hemolysis, reduced oral intake, or presence of paraneoplastic syndromes [14]. In this particular study, we conducted a comprehensive assessment of 95 cancer patients at KKHN to determine the prevalence of anemia in this cohort. Our findings revealed that 50.5% of our patients had anemia across different tumor types. This rate slightly exceeds the figures reported in previous studies conducted within Saudi Arabia (44%) [14], as well as in Turkey (49.7%) [15]. Furthermore, our findings suggest a higher prevalence of anemia compared to a study by Cheng et al., which reported a rate of 18% among Chinese patients [16]. Nevertheless, when comparing our results with available data from Europe and India, our findings indicate a relatively lower prevalence of anemia, with rates of 54.4% [17] and 54.7% [18], respectively. It is important to note that these variations in prevalence rates may stem from inconsistencies in anemia definitions and classifications utilized across studies. Additionally, the inclusion criteria employed in published studies often fail to account for pre-existing anemia or comorbid conditions, thereby contributing to discrepancies in reported rates.

Gastrointestinal cancer (42.1%) emerged as the most prevalent malignancy in our cohort, followed by breast cancer (17.9%). These findings align with two studies from Saudi Arabia and Ethiopia, which reported the highest prevalence of anemia among breast and colorectal cancer patients [14,19]. Wu et al. reported breast cancer (19.5%) as the most common malignancy among anemic cancer patients; however, colorectal cancer (11.9%) was preceded by non-small cell lung cancer (14.9%) [11]. The disparities in prevalence rates observed between our study and these other investigations are minimal and may be attributed to the relatively small sample size in our cohort or, to a greater extent, the higher prevalence of gastrointestinal malignancies in Saudi Arabia [20]. In line with prior reports [14], we observed a higher incidence of anemia among patients with colorectal cancers and urologic genital tract carcinomas. These findings can be anticipated, as disruptions in the normal functioning of the gastrointestinal and urinary tracts may result in asymptomatic blood loss [14,21], However, it is important to note that these associations were not universally observed, as a higher incidence of CIA among patients with lung and ovarian malignancies was also reported [22]. These observations may suggest an underlying inflammatory nature of anemia in cancer patients, which may be suggested by the significantly high rates of ACD in our cohort. While inflammation appears to be a leading cause of anemia, it is crucial to acknowledge that alternative etiologies, such as hemolysis, bone marrow suppression, or malnutrition, may influence the progression of anemia [14]. Furthermore, the majority of cases in our cohort exhibited blood profiles indicative of hypochromic (49%) and microcytic (52%) anemias. These findings align with the observations of Kifle et al. [19], while a Chinese study reported a higher incidence of normocytic anemias (68.6%) [23]. Collectively, these lines of evidence serve to bolster our earlier assertion regarding the multifactorial nature of anemia in cancer patients. These findings indicate that anemia in this population arises from a complex interplay of various mechanisms, contributing to its onset and progression.

Our analysis revealed several significant findings regarding the differences between older (≥65 years) and younger cancer patients (<65 years). Older patients had a higher frequency of having a history of anemia at admission, being in palliative care, having a poor ECOG performance status, requiring increased blood transfusions, and experiencing lower Hb levels during treatment compared to younger patients. These results are consistent with previous studies conducted in Saudi Arabia [14,24]. It appears that older patients exhibit a greater propensity to possess compromised renal function, chronic comorbidities, and overall poorer health status, which renders them more susceptible to an increased risk of CIA. Furthermore, molecular and genomic alterations have been observed among this age group [25].

In our study, we found that Hb levels during treatment were categorized as life-threatening (<6.5 g/dL) in 7.4% of cases, severe (6.5-8 g/dL) in 32.6% of cases, moderate (8-10 g/dL) in 30.5% of cases, and above 10 g/dL in 29.5% of cases. Low ferritin levels were detected in 77% of cases, and 79% of patients required blood transfusions during treatment. These findings are consistent with patterns of anemia severity reported in the literature. For instance, a European study found that almost two-thirds of cancer patients had Hb levels of ≤10 g/dL and 76% of patients required regular blood transfusions [26]. Similarly, Kifle et al. documented mild to moderate anemia in 83.5% of patients [19]. It is worth noting that the consensus regarding the criteria for initiating therapy and stratifying patients with CIA is lacking. As a result, subjectivity may be observed among different healthcare providers and within different healthcare settings.

The factors that predict anemia among cancer patients remain a subject of debate [27]. The existing literature includes retrospective studies with varying variables, which contributes to the lack of solid criteria and limited statistical analysis. Consequently, there is inconsistency in the literature regarding independent prognostic factors associated with anemia in cancer patients. Various factors have been investigated and found to be associated with lower Hb levels, such as advanced stages of cancer (e.g., stage IV), malnutrition, chemotherapy dose and regimen, and patient-specific factors like age and BMI [28,29]. Furthermore, a study by Kifle et al. demonstrated a statistically significant association between anemia occurrence and factors such as ECOG performance score and bleeding history [19]. In our study, we found that older age, an increased number of chemotherapy cycles, and the presence of ACD were associated with the severity of anemia during treatment and were statistically significant. The discrepancy observed in the literature may be attributed, at least in part, to the relatively small sample size of our study (95 patients).

Our study is subject to several limitations that warrant acknowledgment. Firstly, the sample size was relatively small as it was obtained from a single center in Najran City, which restricted the number of patients included in the study to 95 cases. This limited sample size may have influenced the generalizability of our findings to a larger population. Secondly, we did not compare different treatment groups, such as those receiving iron therapy, blood transfusions, or darbepoetin alpha, which could have provided additional insights into the management of anemia in cancer patients. Additionally, certain relevant factors, including predisposing factors and industrial exposures, were not incorporated into our analysis due to the nature and scope of our study. These unaddressed factors may have influenced the occurrence and severity of anemia in our patient cohort. To overcome these limitations and enhance the validity and generalizability of our findings, we recommend the implementation of prospective, larger studies, with clearly identified inclusive and exclusive criteria.

## Conclusions

The present study revealed a notable prevalence of anemia (50.5%) among cancer patients undergoing active treatment, particularly in the context of urologic and gastrointestinal cancers. Factors associated with the severity of anemia included advanced age, an increased number of chemotherapy cycles, and the presence of ACD. Notably, patients aged 65 years and older exhibited a higher frequency of anemia, a poorer ECOG performance status, more Hb decrease levels during treatment, and an increased requirement for blood transfusions. Furthermore, our analysis indicated a positive association between the number of chemotherapy cycles and the need for blood transfusion.

## References

[1] Bray F, Ferlay J, Soerjomataram I, Siegel RL, Torre LA, Jemal A (2018). Global cancer statistics 2018: GLOBOCAN estimates of incidence and mortality worldwide for 36 cancers in 185 countries. CA Cancer J Clin.

[2] Ferlay J, Shin HR, Bray F, Forman D, Mathers C, Parkin DM (2010). Estimates of worldwide burden of cancer in 2008: GLOBOCAN 2008. Int J Cancer.

[3] Alghamdi AH, Niyaz RI, Al-Jifree H, Khan MA, Alsalmi L (2021). Prevalence of anemia among gynecologic cancer patients who received chemotherapy, radiotherapy, or a combination of both at King Abdulaziz Medical City, Jeddah. Cureus.

[4] Cronin KA, Scott S, Firth AU (2022). Annual report to the nation on the status of cancer, part 1: National cancer statistics. Cancer.

[5] Wohlfarth P, Staudinger T, Sperr WR (2014). Prognostic factors, long-term survival, and outcome of cancer patients receiving chemotherapy in the intensive care unit. Ann Hematol.

[6] Schirrmacher V (2019). From chemotherapy to biological therapy: a review of novel concepts to reduce the side effects of systemic cancer treatment (review). Int J Oncol.

[7] Caro JJ, Salas M, Ward A, Goss G (2001). Anemia as an independent prognostic factor for survival in patients with cancer: a systemic, quantitative review. Cancer.

[8] Achariyapota V, Benjapibal M, Chaopotong P (2010). Prevalence and incidence of anemia in Thai patients with gynecologic cancer. Asian Pac J Cancer Prev.

[9] Razzaghdoust A, Mofid B, Peyghambarlou P (2020). Predictors of chemotherapy-induced severe anemia in cancer patients receiving chemotherapy. Support Care Cancer.

[10] Xu H, Xu L, Page JH, Cannavale K, Sattayapiwat O, Rodriguez R, Chao C (2016). Incidence of anemia in patients diagnosed with solid tumors receiving chemotherapy, 2010-2013. Clin Epidemiol.

[11] Wu Y, Aravind S, Ranganathan G, Martin A, Nalysnyk L (2009). Anemia and thrombocytopenia in patients undergoing chemotherapy for solid tumors: a descriptive study of a large outpatient oncology practice database, 2000-2007. Clin Ther.

[12] Aapro MS, Bohlius J, Cameron DA (2011). 2010 update of EORTC guidelines for the use of granulocyte-colony stimulating factor to reduce the incidence of chemotherapy-induced febrile neutropenia in adult patients with lymphoproliferative disorders and solid tumours. Eur J Cancer.

[13] Escobar Álvarez Y, de Las Peñas Bataller R, Perez Altozano J (2021). SEOM clinical guidelines for anaemia treatment in cancer patients (2020). Clin Transl Oncol.

[14] Almehmadi M, Salih M, Elmissbah TE (2021). Prevalence of anemia among Saudi patients with solid cancers at diagnosis in King Faisal Hospital, Taif Province, Kingdom of Saudi Arabia. PLoS One.

[15] Kenar G, Köksoy EB, Ürün Y, Utkan G (2020). Prevalence, etiology and risk factors of anemia in patients with newly diagnosed cancer. Support Care Cancer.

[16] Cheng K, Zhao F, Gao F (2012). Factors potentially associated with chemotherapy-induced anemia in patients with solid cancers. Asian Pac J Cancer Prev.

[17] Ludwig H, Van Belle S, Barrett-Lee P (2004). The European Cancer Anaemia Survey (ECAS): a large, multinational, prospective survey defining the prevalence, incidence, and treatment of anaemia in cancer patients. Eur J Cancer.

[18] Bahl A, Sharma DN, Basu J, Rath GK, Julka PK (2008). Pre-treatment anemia evaluation in cancer patients attending radiotherapy clinic: results from a single Indian center. Indian J Med Sci.

[19] Kifle E, Hussein M, Alemu J, Tigeneh W (2019). Prevalence of anemia and associated factors among newly diagnosed patients with solid malignancy at Tikur Anbessa Specialized Hospital, Radiotherapy Center, Addis Ababa, Ethiopia. Adv Hematol.

[20] Aseafan M, Devol E, AlAhwal M, Souissi R, Sindi R, AlEid H, Bazarbashi S (2022). Population-based survival for cancer patients in Saudi Arabia for the years 2005-2009. Sci Rep.

[21] Chen C, Hu L, Li X, Hou J (2017). Preoperative anemia as a simple prognostic factor in patients with urinary bladder cancer. Med Sci Monit.

[22] Madeddu C, Gramignano G, Astara G, Demontis R, Sanna E, Atzeni V, Macciò A (2018). Pathogenesis and treatment options of cancer related anemia: perspective for a targeted mechanism-based approach. Front Physiol.

[23] Gao F, Cheng K, Zhao F (2011). Prevalence and characteristics of anemia in patients with solid cancers at diagnosis in southwest China. Asian Pac J Cancer Prev.

[24] Bryer E, Henry D (2018). Chemotherapy-induced anemia: etiology, pathophysiology, and implications for contemporary practice. Int J Clin Transfus.

[25] Hung N, Shen CC, Hu YW (2015). Risk of cancer in patients with iron deficiency anemia: a nationwide population-based study. PLoS One.

[26] Ludwig H, Aapro M, Bokemeyer C (2014). A European patient record study on diagnosis and treatment of chemotherapy-induced anaemia. Support Care Cancer.

[27] Bhattathiri VN (2001). Relation of erythrocyte and iron indices to oral cancer growth. Radiother Oncol.

[28] Enkobahry A, Sime T, Kene K, Mateos T, Dilnesa S, Zawdie B (2023). Blood biomarkers as potential malnutrition screening alternatives among adult patients with cancer on treatment in oncology unit of jimma tertiary hospital: a cross-sectional analysis. BMC Nutr.

[29] Muthanna FM, Karuppannan M, Abdulrahman E, Uitrakul S, Rasool BA, Mohammed AH (2022). Prevalence and associated factors of anemia among breast cancer patients undergoing chemotherapy: a prospective study. Adv Pharmacol Pharm Sci.

